# Thromboxane A_2_ receptors contribute to the exaggerated exercise pressor reflex in male rats with heart failure

**DOI:** 10.14814/phy2.15052

**Published:** 2021-09-23

**Authors:** Alec L. E. Butenas, Korynne S. Rollins, Auni C. Williams, Shannon K. Parr, Stephen T. Hammond, Carl J. Ade, K. Sue Hageman, Timothy I. Musch, Steven W. Copp

**Affiliations:** ^1^ Department of Kinesiology Kansas State University Manhattan Kansas USA; ^2^ Department of Anatomy and Physiology Kansas State University Manhattan Kansas USA

**Keywords:** blood pressure, cyclooxygenase, mechanoreflex, muscle afferents, sensory neurons

## Abstract

Mechanical and metabolic signals associated with skeletal muscle contraction stimulate the sensory endings of thin fiber muscle afferents and produce reflex increases in sympathetic nerve activity and blood pressure during exercise (i.e., the exercise pressor reflex; EPR). The EPR is exaggerated in patients and animals with heart failure with reduced ejection fraction (HF‐rEF) and its activation contributes to reduced exercise capacity within this patient population. Accumulating evidence suggests that the exaggerated EPR in HF‐rEF is partially attributable to a sensitization of mechanically activated channels produced by thromboxane A_2_ receptors (TxA_2_‐Rs) on those sensory endings; however, this has not been investigated. Accordingly, the purpose of this investigation was to determine the role played by TxA_2_‐Rs on the sensory endings of thin fiber muscle afferents in the exaggerated EPR in rats with HF‐rEF induced by coronary artery ligation. In decerebrate, unanesthetized rats, we found that injection of the TxA_2_‐R antagonist daltroban (80 μg) into the arterial supply of the hindlimb reduced the pressor response to 30 s of electrically induced 1 Hz dynamic hindlimb muscle contraction in HF‐rEF (*n* = 8, peak ∆MAP pre: 22 ± 3; post: 14 ± 2 mmHg; *p* = 0.01) but not sham (*n* = 10, peak ∆MAP pre: 13 ± 3; post: 11 ± 2 mmHg; *p* = 0.68) rats. In a separate group of HF‐rEF rats (*n* = 4), we found that the systemic (intravenous) injection of daltroban had no effect on the EPR (peak ΔMAP pre: 26 ± 7; post: 25 ± 7 mmHg; *p* = 0.50). Our data suggest that TxA_2_‐Rs on thin fiber muscle afferents contribute to the exaggerated EPR evoked in response to dynamic muscle contraction in HF‐rEF.

## Graphical abstract

We demonstrate that thromboxane A_2_ receptor signaling within the sensory endings of thin fiber muscle afferents of rats with heart failure with reduced ejection fraction (HF‐rEF) contributes to an exaggerated exercise pressor reflex (EPR) evoked during dynamic hindlimb skeletal muscle contractions. Our data may have important clinical implications for our understanding of exercise intolerance in HF‐rEF patients given that activation of the EPR in this patient population contributes to their reduced exercise capacity.

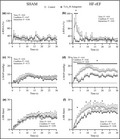

## 1 INTRODUCTION

Heart failure with reduced ejection fraction (HF‐rEF) may develop following myocardial damage and result in systemic manifestations that include increased sympathetic nervous system activity (SNA) at rest (Leimbach et al., 1986; Roveda et al., 2003) and during exercise (Murai et al., 2009; Notarius et al., 1999), as well as reduced exercise capacity (Poole et al., 2012). The elevated SNA during exercise in heart failure contributes importantly to the exercise intolerance within this patient population (Amann et al., 2014). While the mechanisms underlying the exaggerated sympathetic responses to exercise in HF‐rEF patients are complex and multifaceted, a significant body of literature indicates a key role for the exercise pressor reflex (EPR) (Sinoway & Li, 2005). The EPR is activated when the sensory endings of group III and IV thin fiber muscle afferents are stimulated by mechanical and/or metabolic signals associated with skeletal muscle contraction (Kaufman et al., 1982, 1983, 1984; McCloskey & Mitchell, 1972), and in healthy populations, contributes importantly to appropriate increases in SNA, heart rate (HR) and blood pressure (BP) (Amann et al., 2010, 2011; Grotle et al., 2020; Kaufman & Forster, 1996; Kaufman et al., 1983; McCloskey & Mitchell, 1972; Mitchell et al., 1983; Smith, Joyner, et al., 2020; Strange et al., 1993). In contrast to healthy populations, the EPR is exaggerated in HF‐rEF, and its activation impairs oxygen delivery to contracting skeletal muscles (Amann et al., 2014; Ives et al., 2016; Kaur et al., 2018; O'Leary et al., 2004; Smith, Joyner, et al., 2020), thereby reducing exercise capacity in this patient population (Smith, Joyner, et al., 2020). Thus, there is pressing need to investigate the mechanisms of exaggerated EPR activation in HF‐rEF patients.

The mechanically sensitive portion of the EPR (i.e., the mechanoreflex) contributes importantly to the overall EPR exaggeration in HF‐rEF (Butenas et al., 2020; Koba et al., 2008; Middlekauff et al., 2001; Morales et al., 2012; Smith et al., 2005). Our laboratory has used a dynamic rat hindlimb skeletal muscle stretch experimental model, adapted from Stebbins et al. (1988); (Daniels et al., 2000), to study the mechanisms of mechanoreflex activation isolated from contraction‐induced metabolic influence (Butenas et al., 2019; Kempf et al., 2018; Rollins et al., 2019, 2020; Sanderson et al., 2019). This dynamic mechanoreflex activation model in which hindlimb skeletal muscles are stretched at a 1 Hz frequency for 30 s replicates the rhythmic nature of the mechanical stimulus present during locomotor skeletal muscle contractions. Using this model, we found recently that the reflex increases in renal sympathetic nerve activity (RSNA), BP, and HR in response to muscle stretch were greater in rats with HF‐rEF compared with sham‐operated healthy control rats (Butenas, Rollins, et al., 2021). Moreover, we found that blockade of thromboxane A_2_ receptors (TxA_2_‐Rs), a key receptor for cyclooxygenase (COX) metabolites, on sensory endings of thin fiber muscle afferents reduced the sympathetic and cardiovascular responses to hindlimb skeletal muscle stretch in rats with HF‐rEF but not in healthy rats (Butenas, Rollins, et al., 2021). We also reported recently that skeletal muscle COX‐2 isoform protein expression, but not skeletal muscle COX‐1 isoform or dorsal root ganglia TxA_2_‐R protein expression, was greater in HFr‐EF rats than in healthy control rats. These findings collectively suggest that TxA_2_‐R signaling contributes importantly to a chronic or persistent sensitization of the mechanically activated channels that underlie dynamic/rhythmic mechanoreflex activation; an effect that is most likely attributable to HF‐rEF‐induced elevations in COX‐2 metabolites within skeletal muscles. That conclusion built upon a significant body of prior work establishing a role for COX signaling in the exaggerated EPR and mechanoreflex in HF‐rEF patients (Antunes‐Correa et al., 2014; Middlekauff et al., 2008; Morales et al., 2012; Smith, Hart, et al., 2020) and animals (Morales et al., 2012).

Several critical extensions of our recent work summarized above (Butenas, Rollins, et al., 2021) remains to be investigated. First, although the hindlimb muscle stretch model of mechanoreflex activation may provide valuable information regarding the presence of chronic mechanoreflex sensitization in rats with HF‐rEF, the model activates mechanically activated channels in a nonphysiological manner. As such, we sought to determine whether the exaggerated sympathetic and cardiovascular responses to 1 Hz hindlimb muscle stretch in rats with HF‐rEF translated to exaggerated responses to dynamic muscle contraction which produces physiological mechanoreflex activation consequent to muscle shortening and concurrent increases in intramuscular pressure (Gallagher et al., 2001). Second, whether the role for TxA_2_‐Rs in producing the exaggerated responses to 1 Hz muscle stretch extends to experiments evoking 1 Hz muscle contraction is unknown. Lastly, whether HF‐rEF‐induced elevations in skeletal muscle COX‐2 protein expression translate into elevated basal levels of COX metabolites within the skeletal muscle interstitial space remains unknown. Accordingly, we tested the following hypotheses in decerebrate, unanesthetized rats: (1) 30 s of 1 Hz dynamic hindlimb skeletal muscle contraction would result in greater reflex increases in RSNA, mean arterial pressure (MAP), and HR in HF‐rEF rats compared to sham‐operated (SHAM) healthy control rats, (2) injection of the TxA_2_‐R antagonist daltroban (80 µg) into the arterial supply of the hindlimb would reduce the contraction‐induced reflex increases in RSNA, MAP, and HR in HF‐rEF rats but not SHAM rats, and (3) basal skeletal muscle interstitial concentrations of the COX metabolites prostaglandin E_2_ (PGE_2_) and TxB_2_ (a stable derivative of TxA_2_) would be greater in HF‐rEF rats compared with SHAM rats.

## 2 METHODS AND MATERIALS

### 2.1 Ethical approval

All experimental procedures were approved by the Institutional Animal Care and Use Committee of Kansas State University and conducted in accordance with the National Institutes of Health Guide for the Care and Use of Laboratory Animals (2011). Experiments were performed on ~14‐ to 19‐week‐old male Sprague–Dawley rats (*n* = 60; Charles River Laboratories). Rats were housed two per cage in temperature (maintained at ~22℃) and light (12–12 h light–dark cycle running from 7 a.m. to 7 p.m.)‐controlled accredited facilities with standard rat chow and water provided ad libitum.

### 2.2 Surgical procedure

Myocardial infarction (MI) was induced in 29 of the 60 rats by surgically ligating the left main coronary artery (Musch & Terrell, 1992). Briefly, rats were anesthetized initially with a 5% isoflurane‐O_2_ mixture (Butler Animal Health Supply, and Linweld) and maintained subsequently on 2.5% isoflurane‐O_2_ and then intubated and mechanically ventilated with a rodent respirator (Harvard model 680, Harvard Instruments) for the duration of the surgical procedure. After a single injection of amiodarone (100 mg/kg ip), a left thoracotomy was performed to expose the heart through the fifth intercostal space, and the left main coronary artery was ligated 1–2 mm distal to the edge of the left atrium with a 6‐0 braided polyester suture. The thorax was then closed with 2‐0 gut, and the skin was closed with 2‐0 silk. Prior to termination of anesthesia, bupivacaine (1.5 mg/kg sc) and buprenorphine (~0.03 mg/kg im) were administered to reduce pain associated with the surgery, along with ampicillin (50 mg/kg im) to reduce the risk of infection. After rats were removed from mechanical ventilation and anesthesia, they were monitored closely for ~6 h post‐surgery. In the remaining 31 of 60 rats, a sham ligation of the coronary artery was performed in which 6‐0 braided polyester suture was passed under the left main coronary artery, but not tied. These rats are referred to as “SHAM” rats from this point forward. Following completion of either MI or SHAM procedures, rats were housed one per cage for 10 days to minimize risk of infection of the surgical site. During these 10 days, the antibiotic baytril (100 mg/ml) was administered in the drinking water. Following completion of the baytril treatment, rats were housed two per cage as described above. All animals were monitored daily for 14 days following MI or SHAM procedure for changes in behavior, gait/posture, breathing, appetite, and body weight.

### 2.3 Echocardiograph measurements

Transthoracic echocardiograph measurements were performed with a commercially available system (Logiq S8; GE Health Care) no more than 1 week before the final experimental protocol. Briefly, the rats were anaesthetized as described above. Once the rat was fully anesthetized, the isoflurane mixture was reduced to 2.5% isoflurane‐O_2_. Following 5 min at 2.5% isoflurane, echocardiograph measurements began. The transducer was positioned on the left anterior chest, and left ventricular dimensions were measured. The left ventricular fractional shortening (FS), ejection fraction (EF), end diastolic volume (LVEDV), end systolic volume (LVESV), and stroke volume (SV) were determined by echocardiographic measurements as previously described (Baumfalk et al., 2021). Rats with HF‐rEF were required to meet an inclusion criterion of either an FS ≤30% and/or a left ventricular infarct size of ≥15%, which is consistent with values previously reported from our laboratory (Butenas, Colburn, et al., 2021; Butenas et al., 2020; Butenas, Rollins, et al., 2021). We have previously shown that, compared with SHAM‐operated healthy control rats, HF‐rEF rats that meet similar criterion have a reduced maximal oxygen uptake and time to exhaustion to treadmill running (Butenas, Colburn, et al., 2021).

### 2.4 Surgical procedures for experimental protocols

In vivo experiments were performed on 34 rats (15 SHAM, 19 HF‐rEF) between 6 and 8 weeks following the MI or SHAM procedure. On the day of the experiment, rats were anesthetized as described above. Adequate depth of anesthesia was confirmed by the absence of toe‐pinch and blink reflexes. The trachea was cannulated, and the lungs were mechanically ventilated (Harvard Apparatus) with a 2% isoflurane‐balance O_2_ gaseous mixture until the decerebration was completed (see below). The right jugular vein and both carotid arteries were cannulated with PE‐50 catheters which were used for the injection of fluids, measurement of arterial BP (physiological pressure transducer, AD Instruments), and sampling of arterial blood gasses (Radiometer). HR was calculated from the R‐R interval measured by electrocardiogram (AD Instruments). The left superficial epigastric artery was cannulated with a PE‐8 catheter whose tip was placed near the junction of the superficial epigastric and femoral arteries. A reversible snare was placed around the left iliac artery and vein (i.e., proximal to the location of the catheter placed in the superficial epigastric artery). The left calcaneal bone was severed and linked by string to a force transducer (Grass FT03), which, in turn, was attached to a rack and pinion. An ~1–2 cm section of the left sciatic nerve was exposed by reflecting back the overlaying skeletal muscles.

Upon completion of the initial surgical procedures, rats were placed in a Kopf stereotaxic frame. After administering dexamethasone (0.2 mg i.v.) to minimize swelling of the brainstem, a pre‐collicular decerebration was performed in which all brain tissue rostral to the superior colliculi was removed (Smith et al., 2001). Following decerebration, anesthesia was reduced to 0.5%. A retroperitoneal approach was used to expose bundles of the left renal sympathetic nerve, which were then glued (Kwik‐Sil, World Precision Instruments) onto a pair of thin stainless‐steel recording electrodes connected to a high impedance probe (Grass Model HZP) and amplifier (Grass P511). Multiunit signals from the renal sympathetic nerve fibers were filtered at high and low frequencies (1 kHz and 100 Hz, respectively) for the measurement of RSNA. Successful RSNA recordings were made in 13 SHAM rats and 13 HF‐rEF rats.

Upon completion of all surgical procedures, anesthesia was terminated, and the rats’ lungs were ventilated with room air. Experimental protocols commenced at least 1 h after reduction of isoflurane from 2% to 0.5%, and at least 30 min after reduction of isoflurane from 0.5% to 0%. Experiments were performed on decerebrate, unanesthetized rats because anesthesia has been shown to markedly blunt the EPR in the rat (Smith et al., 2001). Body core temperature was measured via a rectal probe and maintained at ~37–38℃ by an automated heating system (Harvard Apparatus) and heat lamp. Arterial pH and blood gases were analyzed periodically throughout the experiment from arterial blood samples (~75 μL) and maintained within physiological ranges (pH: 7.35–7.45, PCO_2_: ~38–40 mmHg, PO_2_: ~100 mmHg) by administration of sodium bicarbonate and/or adjusting ventilation as necessary. At the end of all experiments in which RSNA was measured, postganglionic sympathetic nerve activity was abolished with administration of hexamethonium bromide (20 mg/kg i.v.) to allow for the quantification of background noise as described previously (Kempf et al., 2018). Rats were then anaesthetized with 5% isoflurane and humanely euthanized with an injection of potassium chloride (>3 mg/kg i.a). A pneumothorax was then performed, and the heart was excised. The atria and right ventricle (RV) were separated from the left ventricle (LV) and septum, and the RV, LV, and atria were weighed. In rats with HF‐rEF, the LV infarction surface area was measured using planimetry and expressed as percent of LV endocardial surface area as described previously (Craig et al., 2019).

### 2.5 EPR protocols

In 34 rats (15 SHAM, 19 HF‐rEF), we studied the EPR evoked in response to 1 Hz dynamic hindlimb muscle contraction. Following recovery of isoflurane anesthesia, baseline muscle tension was set to ~100 g and baseline RSNA, BP, and HR were measured for ~30 s. The sciatic nerve was then electrically stimulated using stainless‐steel electrodes for 30 s at a voltage of ~1.5x motor threshold (0.01 ms pulse duration, 500 ms train duration, 40 Hz frequency) which produced 1 Hz repetitive/dynamic contractions of the triceps surae muscles. In 18 of these 35 rats (10 SHAM, 8 HF‐rEF) we investigated the effect of TxA_2_‐R blockade on the EPR. In these 18 rats, ~10 min following the control contraction maneuver, the snare on the left iliac artery and vein was tightened and the TxA_2_‐R antagonist daltroban (Santa Cruz Biotechnology, Inc.; 80 µg dissolved in 0.4 ml of 1% DMSO/saline solution; Leal et al., 2011) was injected into the arterial supply of the hindlimb through the superficial epigastric artery catheter. Daltroban remained snared in the hindlimb circulation for 5 min, at which time the iliac snare was released. The hindlimb was reperfused for 10 min and the dynamic contraction maneuver was then repeated exactly as described above. At the end of all experiments, we injected the paralytic pancuronium bromide (1 mg/kg i.v.) and the sciatic nerve was stimulated for 30 s with the same parameters as those used to elicit contraction to ensure that the increase in RSNA, BP, and HR during contraction was not due to the electrical activation of the axons of the thin fiber muscle afferents in the sciatic nerve. No increase in RSNA, BP, or HR, were observed during the stimulation period following the administration of pancuronium bromide. Additionally, at the end of each experiment Evans blue dye was injected in the same manner as the experimental solution to confirm that the injectate had access to the triceps surae muscle circulation. The triceps surae muscles were observed to stain blue in all experiments.

### 2.6 Control experiments

In four of the 19 HF‐rEF rats in which the EPR was evoked, we investigated whether 1% DMSO (i.e., the vehicle for daltroban) or a systemic circulation of daltroban may have accounted for the attenuating effects in the main experimental group in which daltroban was injected into the hindlimb arterial circulation. In these four rats, the DMSO and the intravenous injection protocols were performed in series with ~10 min recovery time between protocols. The 1% DMSO protocols were performed exactly as described above for the daltroban experiments except 0.4 ml of 1% DMSO alone was injected into the arterial supply of the hindlimb via the left superficial epigastric artery catheter. The systemic circulation control protocols were performed as described above except 80 µg of daltroban was injected into the catheter placed in the jugular vein. The dynamic hindlimb muscle contraction maneuver was then performed 15 min after injection to match the timing of the daltroban injection into the arterial supply of the hindlimb described above.

### 2.7 Muscle microdialysis to measure PGE_2_

Microdialysis experiments were performed on a total of 20 rats (SHAM *n* = 11, HF‐rEF *n* = 9). Briefly, microdialysis probes (LM‐10, BASi Research Products) were connected to a PE‐50 tubing which was attached to an adaptor tip and syringe filled with sterile physiological saline. Immediately following the decerebration and termination of anesthesia, one microdialysis probe was inserted into the white portion of the gastrocnemius muscle with a 20‐gauge hollow needle. At least 1 h of recovery was allowed following probe placement before beginning the microdialysis protocol. Following the 1 h recovery period, sterile saline was perfused through the probes at a constant rate of 5 μl/min. Baseline dialysate fluid was collected for 25 min in order to measure baseline concentrations for PGE_2_ and TxB_2_ (i.e., the stable derivative of TxA_2_). Microdialysate fluid was stored immediately after collection at −80℃ freezer until further analysis using the commercially available ELISA kits for PGE_2_ (Cayman Chemical; Item no. 514010) and TxB_2_ (Cayman Chemical; Item no. 501020).

### 2.8 COX‐2 activity analysis

Enzyme activity experiments were performed on a sample of nine rats (4 SHAM, 5 HF‐rEF). A tissue sample (~180 mg) from the white portion of the gastrocnemius muscle was collected from the right hindlimb. Samples were rinsed with 0.1 M TRIS‐Buffer (pH 7.4), and homogenized in 1 ml of 0.1 M TRIS‐HCl, pH 7.8, containing 1 mM EDTA for 1 min at 5 m/s using 2 ml tubes containing ~0.5 g of 1.4 mm ceramic beads using Bead Mill 4 (Fisherbrand^™^). Samples were then centrifuged at 10,000*g* for 15 min at 4℃. The supernatant was removed and analyzed for COX‐2 activity (Cayman Chemical; Item no. 760151) according to manufacturer's instructions. Absorbance was read at 590 nm using accuSkan^™^ FC Filter‐Based Microplate Photometer (Fisherbrand^™^).

### 2.9 Western blot and quantitative reverse transcriptase polymerase chain reaction experiments for TxA_2_‐R expression

In eight rats (4 SHAM, 4 HF‐rEF) the left and right L_4_ and L_5_ DRG were harvested. Samples were isolated into 2 ml bead mill tubes containing ~0.5 g of 1.4 mm ceramic beads and 300 μL of ML lysis buffer (Macherey‐Nagel) and homogenized for 1 min at 5 m/s using Bead Mill 4 (Fisherbrand^™^). Total protein and mRNA from tissues were prepared with the Nucleospin miRNA/Protein Kit (Macherey‐Nagel; Ref. no. 740971.50) according to the manufacturer's instructions. Total Protein and RNA concentrations were determined using the Qubit 2.0 Fluorometer (Life Technologies). Protein samples (40 μg) were separated on 4–12% Bis‐Tris Protein Gels (Invitrogen^™^) by gel electrophoresis in MES running buffer (Invitrogen^™^) employing 220 V for 22 min. Gels were then transferred to mini‐PVDF membranes using the iBlot 2 Dry Transfer Device (Invitrogen^™^). The membrane was incubated for ~3 h with the iBind device with iBind solution (Invitrogen^™^) with the primary antibodies: anti TxA_2_‐R diluted 1:100 (Santa Cruz Biotechnology; cat. no. SC‐515033; RRID:AB_2847878) and loading control antibody anti‐GAPDH diluted 1:1000 (Thermofisher Scientific; cat. no. MA5‐15738; RRID:AB_10977387) as well as the secondary antibody conjugated with Horse Radish Peroxidase diluted 1:1500 (Thermofisher Scientific; cat. no. 31430; RRID:AB_228307). Membranes were then incubated for 5 min with SuperSignal^™^ West Pico PLUS Chemiluminescent Substrate (Thermofisher) and imaged with C‑DiGit^®^ Blot Scanner (Li‐Cor). The protein bands were quantified and analyzed using the Image Studio software (Li‐Cor). Complementary DNA (cDNA) was synthesized from RNA isolates (see above) using the High Capacity RNA‐cDNA^™^ kit (ThermoFisher) according to the manufacturer's instructions as described previously (Copp et al., 2016). Quantitative reverse transcriptase polymerase chain reaction experiments were then performed on the cDNA samples using TaqMan gene expression assays specific for: TxA_2_‐R (Sequence proprietary; Assay ID: Rn00690601_m1), and GAPDH with forward primer: 5′‐ACCGCCTGTTGCGTGTTA‐3′ and reverse primer: 5′‐CAATCGCCAACGCCTCAA‐3′. All samples were run in duplicate for the gene of interest, and the endogenous control (GAPDH). The results were analyzed with the comparative threshold (ΔΔCt) method.

### 2.10 Data analysis

Muscle tension, BP, HR, and RSNA were measured and recorded in real time with a PowerLab and LabChart data acquisition system (AD Instruments). The original RSNA data were rectified and corrected for the background noise determined after the administration of hexamethonium bromide. Baselines for MAP, RSNA and HR were determined from the 30‐s baseline periods that preceded each maneuver. The peak increase in MAP (peak ΔMAP), RSNA (peak ΔRSNA), and HR (peak ΔHR) during dynamic contraction were calculated as the difference between the peak values wherever they occurred during the maneuvers and their corresponding baseline value. The integrated RSNA for the first 5 s (∫∆RSNA_5 s_) was calculated by integrating the ∆RSNA (>0) during the first 5 s of the contraction maneuver. The change in tension‐time indexes (ΔTTIs) and blood pressure indexes (BPIs) was calculated by integration of the area under curve during the contraction maneuver and subtracting the integrated area under the curve during the baseline period. Time courses of the increase in RSNA, MAP and HR were plotted as their change from baseline. Student's *t*‐tests or Mann–Whitney tests were performed to compare baseline MAP, baseline HR, Peak ΔMAP, Peak ΔHR, Peak ΔRSNA, BPI, ∫∆RSNA, and ΔTTI across groups. Šidák multiple comparisons tests were performed for within animal comparisons of those same variables. Data for echocardiograph measurements, body and organ masses, heart morphometrics, protein, and mRNA expression were analyzed with unpaired Student's *t*‐tests or Mann–Whitney *U* tests as appropriate. All data are expressed as mean ± SEM. Statistical significance was defined as *p* ≤ 0.05.

## 3 RESULTS

### 3.1 Body mass and heart morphometrics

Body mass as well as the ratio of LV to body mass were not different between SHAM and HF‐rEF rats (Table 1). The ratios of the RV and atria mass to body mass were higher in HF‐rEF rats compared to SHAM rats. Additionally, LVEDV, LVESV, and SV were significantly higher, while EF and FS were significantly lower, in HF‐rEF rats compared to SHAM rats.

**TABLE 1:** Body and tissue masses and heart morphometrics in SHAM and HF‐rEF rats

	SHAM (*n* = 31)	HF‐rEF (*n* = 29)	*p*‐value
Body mass	526 ± 7	521 ± 7	0.67
LV/body mass (mg/g)	2.02 ± 0.04	2.08 ± 0.04	0.25
RV/body mass (mg/g)	0.51 ± 0.01	0.58 ± 0.02	<0.01*
Atria/body mass (mg/g)	0.16 ± 0.01	0.20 ± 0.01	<0.01*
LV EDV (ml)	1.07 ± 0.06	2.30 ± 0.12	<0.01*
LV ESV (ml)	0.17 ± 0.02	1.23 ± 0.08	<0.01*
Stroke volume (ml)	0.90 ± 0.05	1.08 ± 0.06	0.03*
Ejection fraction (%)	84 ± 1	46 ± 2	<0.01*
Fractional shortening (%)	49 ± 1	21 ± 1	<0.01*
Infarct size (%)	—	28 ± 1	—

Values are means ± SEM. Data were compared using student's *t*‐tests or Mann–Whitney tests as appropriate. Asterisks indicate a statistically significant difference between groups (*p* < 0.05).

Abbreviations: EDV, end diastolic volume; ESV, end systolic volume; HF‐rEF, heart failure with reduced ejection fraction; LV, left ventricle; RV, right ventricle.

### 3.2 Effect of HF‐rEF on the EPR

**FIGURE 1:**
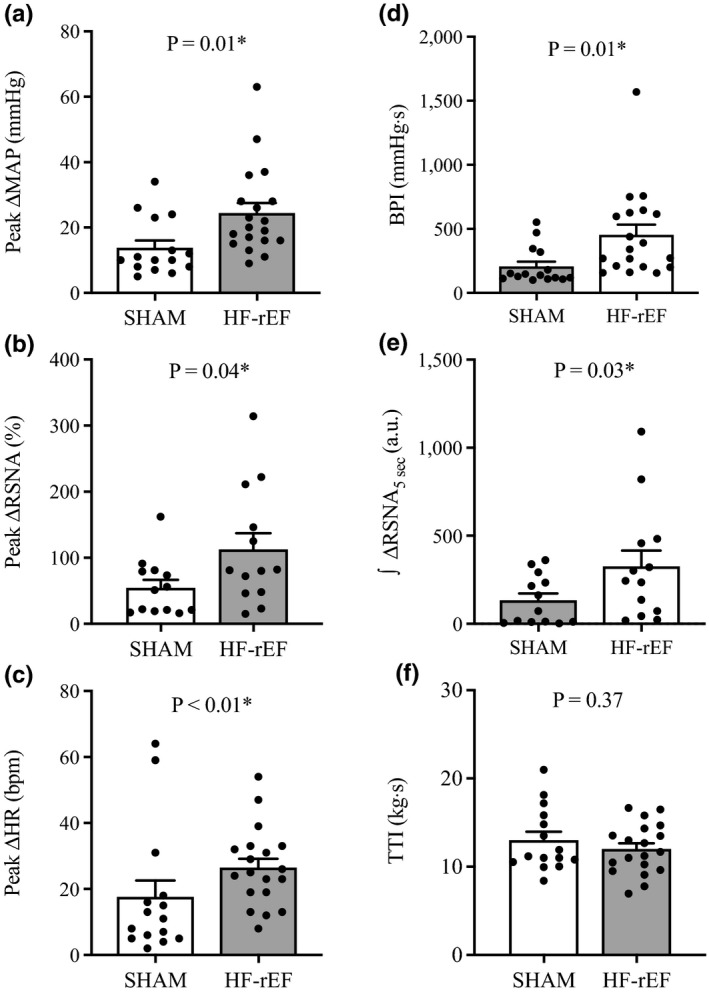
Effect of HF‐rEF on dynamic exercise pressor reflex activation. The peak ∆ mean arterial pressure (MAP; a), peak ∆ renal sympathetic nerve activity (RSNA; b), peak ∆ heart rate (HR; c), blood pressure index (BPI, d), and the first 5 s of the integrated change in RSNA (∫∆RSNA_5 s_, e) in response to 30 s of dynamic hindlimb muscle contraction in SHAM (*n* = 15) and HF‐rEF (*n* = 19) rats. TTI, tension‐time index (f). Data were analyzed with Student's *t*‐tests or Mann–Whitney tests as appropriate and are expressed as mean ± SEM with individual data points. Asterisks indicates statistically significant differences between groups (*p* < 0.05). HF‐rEF, heart failure with reduced ejection fraction

**FIGURE 2:**
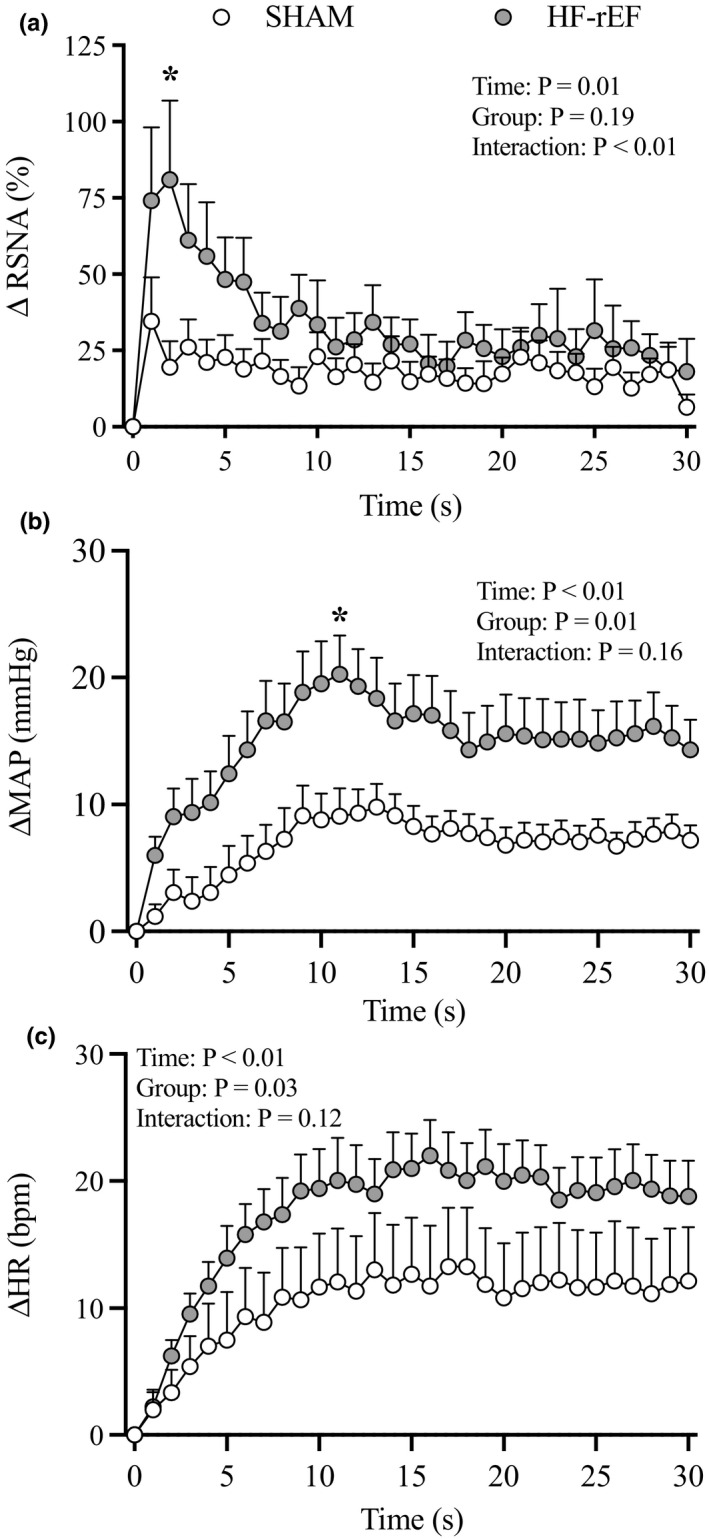
Effect of HF‐rEF on the time course of dynamic exercise pressor reflex activation. The ∆ renal sympathetic nerve activity (RSNA; a), ∆ mean arterial pressure (MAP; b), and ∆ heart rate (HR; c) response to 30 s of dynamic hindlimb muscle contraction in SHAM (*n* = 15) and HF‐rEF (*n* = 19) rats. Data were analyzed with two‐way ANOVAs with Šidák multiple comparisons tests and are expressed as mean ± SEM. Asterisks indicate time points where comparisons were statistically significant (*p* < 0.05). HF‐rEF, heart failure with reduced ejection fraction

**FIGURE 3:**
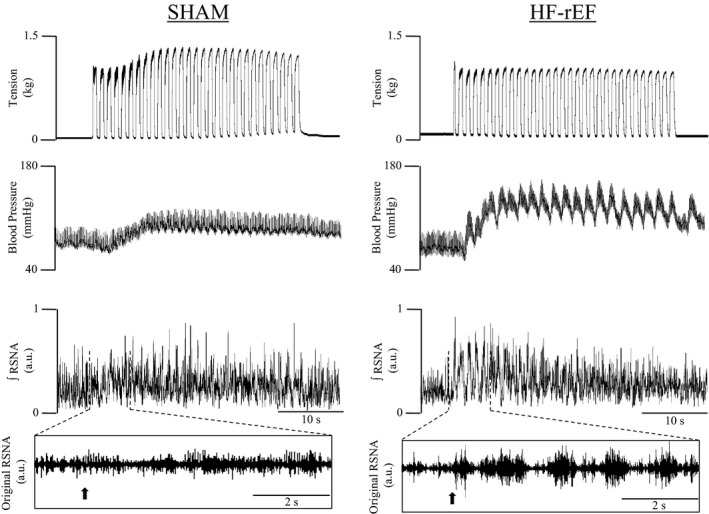
Examples of original tracings of the blood pressure, and integrated renal sympathetic nerve activity (∫RSNA, in arbitrary units [a.u.]) response to 30 s of dynamic hindlimb muscle contraction in a SHAM rat (left) and HF‐rEF rat (right). *Insets*: original renal sympathetic nerve recording. Arrows indicate onset of contraction. HF‐rEF, heart failure with reduced ejection fraction; RSNA, renal sympathetic nerve activity

Dynamic hindlimb muscle contraction evoked larger sympathetic and cardiovascular responses in HF‐rEF rats (*n* = 19) compared with SHAM rats (*n* = 15, Figures 1 and 2). The TTI of the contraction maneuver was not different between groups (Figure 1F). Examples of original tracings of the increase in BP and integrated RSNA in response to 30 s of 1 Hz dynamic hindlimb skeletal muscle contraction from one SHAM rat and one HF‐rEF rat are shown in Figure 3.

### 3.3 Effect of TxA_2_‐R blockade on the EPR

**FIGURE 4:**
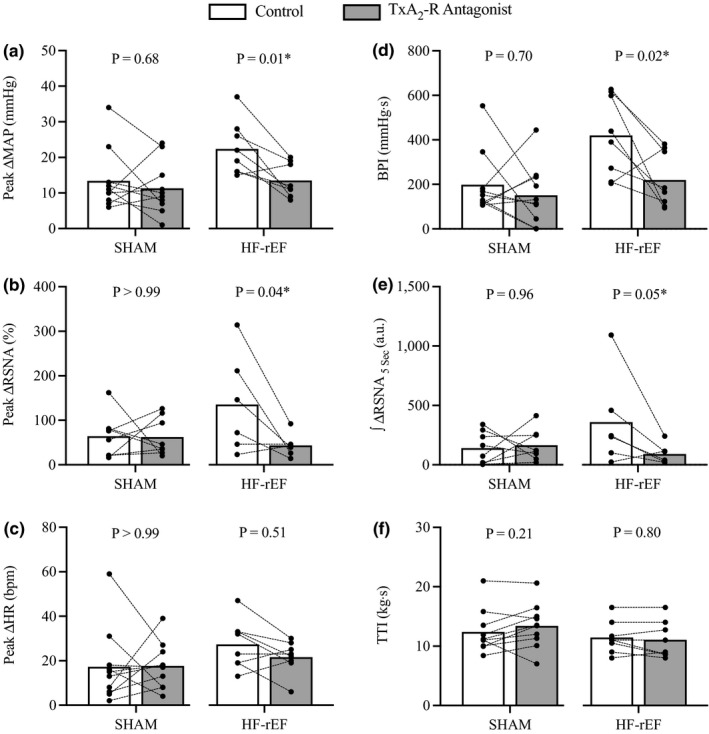
Effect of TxA_2_‐R blockade on dynamic exercise pressor reflex activation. The peak ∆ mean arterial pressure (MAP; a), peak ∆ renal sympathetic nerve activity (RSNA; b), peak ∆ heart rate (HR; c), blood pressure index (BPI, d), and the first 5 s of the integrated change in RSNA (∫∆RSNA_5 s_, e) in response to 30 s of dynamic hindlimb muscle contraction before (Control) and after injection of the TxA_2_‐R antagonist daltroban (80 µg) into the arterial supply of the hindlimb in SHAM (*n* = 10) and HF‐rEF (*n* = 8) rats. TTI, tension‐time index (f). Data were analyzed with Šidák multiple comparisons tests and are expressed as mean ± SEM overlaid with individual responses. Asterisks indicate statistically significant differences between groups (*p* < 0.05). HF‐rEF, heart failure with reduced ejection fraction; TxA_2_‐R, thromboxane A_2_ receptor

**FIGURE 5:**
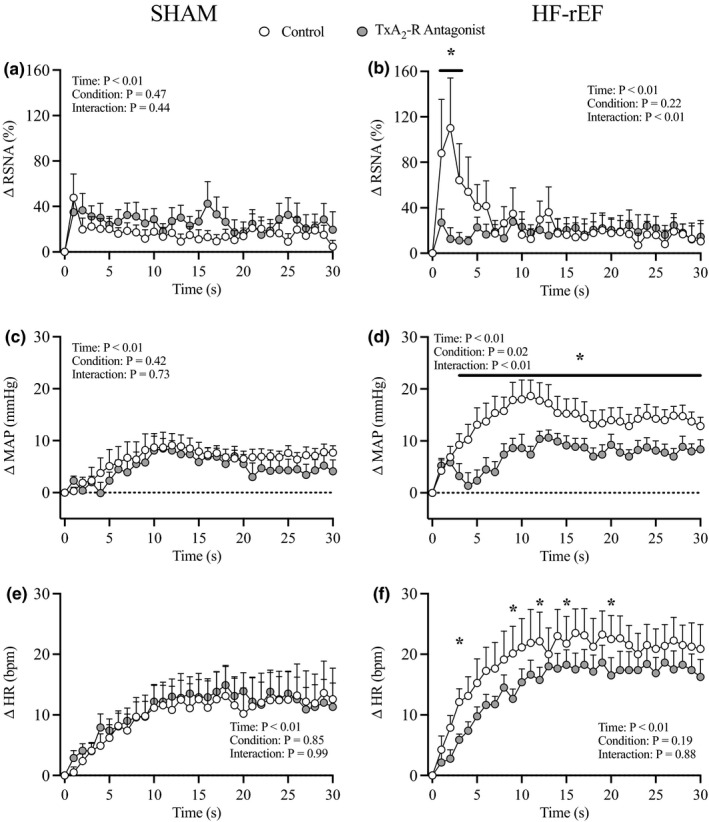
Effect of TxA_2_‐R blockade on the time course of dynamic exercise pressor reflex activation. Δ renal sympathetic nerve activity (RSNA; a, b), Δ mean arterial pressure (MAP; c, d), and Δ heart rate (HR; e, f) during 30 s of dynamic hindlimb muscle contraction before (Control) and after injection of 80 µg of the TxA_2_‐R antagonist into the arterial supply of the hindlimb in SHAM (*n* = 10) and HF‐rEF (*n* = 8) rats. Data were analyzed with two‐way ANOVAs and Šidák multiple comparisons tests and are expressed as mean ± SEM. Asterisks and/or black lines indicate time points where comparisons were statistically significant (*p* < 0.05). HF‐rEF, heart failure with reduced ejection fraction; TxA_2_‐R, thromboxane A_2_ receptor

**FIGURE 6:**
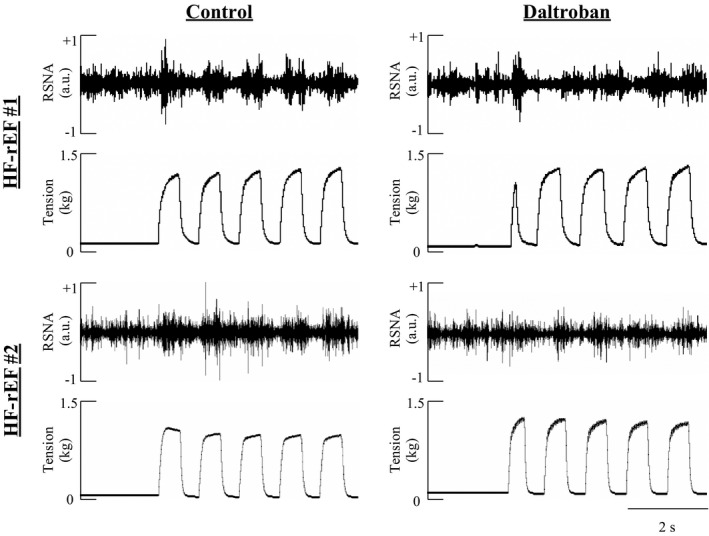
Examples of the distinct renal sympathetic nerve activity (RSNA) bursts that occur with each tension development during 1 Hz dynamic contraction in two HF‐rEF rats before (Control) and after injection of 80 µg of the TxA_2_‐R antagonist into the arterial supply of the hindlimb. The synchronization between RSNA and the tension development in the control condition suggests that dynamic hindlimb muscle contractions primarily provide a mechanical stimulus, which was effectively attenuated following TxA_2_‐R antagonism. HF‐rEF, heart failure with reduced ejection fraction; TxA_2_‐R, thromboxane A_2_ receptor

In SHAM rats (*n* = 10), we found that injection of the TxA_2_‐R antagonist daltroban into the arterial supply of the hindlimb had no effect on the sympathetic and cardiovascular responses to dynamic contraction (Figures 4 and 5). Conversely, in HF‐rEF rats (*n* = 11), injection of daltroban into the arterial supply of the hindlimb significantly reduced the sympathetic and cardiovascular responses to dynamic contraction (Figures 4 and 5). Figure 6 shows original tracings from two HF‐rEF rats of the first 5 s of contraction demonstrating that the RSNA bursts were synchronized with muscle tension development and that the bursts were reduced following TxA_2_‐R blockade. The TTI of the contraction maneuver was not different between control and TxA_2_‐R blockade conditions in either SHAM or HF‐rEF rats (Figure 4F). Baseline MAP and HR were not different between conditions in SHAM or HF‐rEF rats (Table 2; *p* > 0.15 for both).

**TABLE 2:** Baseline MAP and HR in SHAM and HF‐rEF rats

	SHAM		HF‐rEF	
	Control	Post condition	Control	Post condition
Baseline MAP, mmHg				
Daltroban i.a.	85 ± 5	81 ± 5	81 ± 4	84 ± 6
Daltroban i.v.	—	—	87 ± 9	83 ± 10
1% DMSO i.a.	—	—	83 ± 10	86 ± 8
Baseline HR, bpm				
Daltroban i.a.	429 ± 9	438 ± 11	448 ± 18	452 ± 20
Daltroban i.v.	—	—	454 ± 15	462 ± 18
1% DMSO i.a.	—	—	462 ± 18	475 ± 4

Values are means ± SEM. Data were compared between conditions (control vs. post condition) with paired Student's *t*‐tests. There was no significant difference for any comparison.

Abbreviations: HF‐rEF, heart failure with reduced ejection fraction; HR, heart rate; MAP, mean arterial pressure.

### 3.4 Control experiments

In four HF‐rEF rats, we found that 1% DMSO had no effect on the peak ∆MAP (control: 23 ± 8, 1% DMSO: 20 ± 6 mmHg; *p* = 0.40), BPI (control: 463 ± 224, 1% DMSO: 335 ± 133 mmHg·s; *p* = 0.26), or peak ∆HR (control: 27 ± 8, 1% DMSO: 24 ± 6 bpm; *p* = 0.75) response to dynamic contraction. The TTI of the contraction maneuver was not different between control (11 ± 1 kg·s) and 1% DMSO (11 ± 2 kg·s; *p* = 0.57) conditions. Baseline MAP and HR were not different between conditions (Table 2). These findings suggest that the attenuating effect of daltroban produced when it was injected into the arterial supply of the hindlimb of HF‐rEF rats was not attributable to effects produced by its vehicle 1% DMSO.

In the same four HF‐rEF rats used in 1% DMSO experiments, we found that i.v. injection of daltroban had no effect on the peak ∆MAP (control: 26 ± 7, daltroban i.v.: 25 ± 7 mmHg; *p* = 0.50), BPI (control: 362 ± 132, daltroban i.v.: 490 ± 213 mmHg·s; *p* = 0.22), or peak ∆HR (control: 20 ± 4, daltroban i.v.: 28 ± 8 bpm; *p* = 0.28) response to dynamic contraction. The TTI of the contraction maneuver was not different between control (11 ± 2 kg·s) and daltroban i.v. (12 ± 1 kg·s; *p* = 0.65) conditions. Baseline MAP and HR were not different between conditions (Table 2). These findings suggest that the attenuating effect of daltroban produced when it was injected into the arterial supply of the hindlimb of HF‐rEF rats is most likely attributable to blockade of TxA_2_‐R on the sensory endings of thin fiber muscle afferents and not systemic effects elsewhere in the EPR arc such as the brainstem and/or the spinal cord.

### 3.5 Basal skeletal muscle interstitial PGE_2_ and TxB_2_ concentrations and COX‐2 activity

**FIGURE 7:**
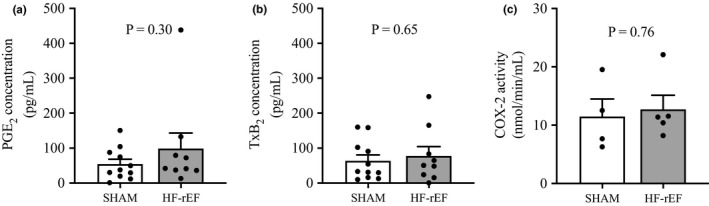
Effect of HF‐rEF on interstial PGE_2_ and TxB_2_ concentrations and COX‐2 activity in the white portion of the gastrocnemius muscle. Basal concentrations of PGE_2_ (a) and TxB_2_ (b) within microdialysate fluid collected from the white portion of the gastrocnemius muscle in SHAM (*n* = 11) and HF‐rEF (*n* = 9) rats. Confirmation of COX‐2 enzymatic activity from muscle homogenates taken from the white portion of the gastrocnemius muscle (c) of a distinct group of SHAM (*n* = 4) and HF‐rEF (*n* = 5) rats. Data were analyzed with Student's *t*‐tests and are expressed as mean ± SEM with individual data points. HF‐rEF, heart failure with reduced ejection fraction; PGE_2_, prostaglandin E_2_

In 11 SHAM rats and nine HF‐rEF rats, we found that baseline microdialysate fluid concentrations from the white portion of the gastrocnemius muscle for PGE_2_ as well as TxB_2_ were not different (Figure 7A,B). Further, in different rats not used in microdialysis experiments (four SHAM and five HF‐rEF) we found no difference in COX‐2 enzymatic activity within skeletal muscle homogenates taken from the white portion of the gastrocnemius muscle (Figure 7C).

### 3.6 DRG TxA_2_‐R protein and mRNA expression

We found no difference between SHAM (*n* = 4) and HF‐rEF (*n* = 4) rats in TxA_2_‐R protein (SHAM: 1.00 ± 0.49; HF‐rEF: 0.72 ± 0.44 a.u.; *p* = 0.60) or mRNA expression (SHAM: 1.00 ± 0.07; HF‐rEF: 0.93 ± 0.09 a.u.; *p* = 0.51) within L_4_ and L_5_ DRG tissue. This was an important reproduction and confirmation of the same findings reported in our recent investigation (Butenas et al., 2020).

## 4 DISCUSSION

The present investigation is an important extension of our previous findings which showed that TxA_2_‐Rs contribute to the exaggerated sympathetic and cardiovascular responses to 1 Hz dynamic hindlimb muscle stretch in rats with HF‐rEF (Butenas, Rollins, et al., 2021). Specifically, we have now extended those previous findings from the muscle stretch model of isolated mechanoreflex activation to a 1 Hz dynamic muscle contraction protocol which produces physiological mechanoreflex activation consequent to muscle shortening and intramuscular pressure development. We found that the increases in RSNA, BP, and HR evoked in response to 1 Hz dynamic hindlimb skeletal muscle contraction were greater in HF‐rEF rats than the increases observed in healthy SHAM rats. We also found that hindlimb arterial injection of an antagonist for TxA_2_‐Rs reduced the exaggerated responses to hindlimb muscle contraction in HF‐rEF rats, whereas there was no effect of TxA_2_‐R antagonism in SHAM rats. In contrast to our hypothesis, we found no difference between SHAM and HF‐rEF rats in resting hindlimb skeletal muscle (white portion of the gastrocnemius) interstitial concentrations for two primary agonists for TxA_2_‐Rs (i.e., PGE_2_ and TxB_2_) as well as no difference between SHAM and HF‐rEF rats in basal COX‐2 enzymatic activity from skeletal muscle homogenates of the same muscle region. Collectively, our findings suggest that TxA_2_‐Rs on the sensory endings of thin fiber muscle afferents contribute to the exaggerated EPR in rats with HF‐rEF, but that the effect does not appear attributable to augmented basal concentrations of COX metabolites within hindlimb skeletal muscles.

We used a 1 Hz dynamic hindlimb muscle contraction maneuver designed to closely replicate the frequency of muscle contractions during locomotion. This contraction maneuver presents the sensory endings of thin fiber muscle afferents with a robust mechanical stimulus as evidenced by the synchronization of RSNA bursting with skeletal muscle tension development (Figures 3 and 6, as well as refs: Copp et al., 2016; Kempf et al., 2018; Victor et al., 1988), as well as by the similar increases in RSNA and BP during 1 Hz dynamic stretch compared to contraction (Kempf et al., 2018). A metabolic stimulus to the sensory endings of thin fiber muscle afferents during the maneuver is also possible but presumably significantly less important than the mechanical stimulus. Indeed, dynamic hindlimb muscle contraction as evoked in the present investigation markedly increases hindlimb skeletal muscle blood flow (Copp et al., 2015) which likely minimizes metabolite accumulation within the hindlimb, especially when compared to a static hindlimb muscle contraction maneuver during which blood flow is reduced compared to rest and metabolites likely accumulate to a substantial degree (Ducrocq et al., 2020; Grotle et al., 2019). Thus, we believe the dynamic hindlimb muscle contraction maneuver is an excellent experimental model that can be used to study the EPR with substantial contribution from the mechanoreflex. In this regard, our present findings that the sympathetic and cardiovascular responses to dynamic muscle contraction were exaggerated in HF‐rEF rats compared to SHAM rats builds upon our previous findings which showed that the sympathetic and cardiovascular responses to 1 Hz dynamic skeletal muscle stretch were exaggerated in HF‐rEF rats compared to SHAM rats (Butenas, Rollins, et al., 2021). Our findings are also consistent with reports of greater sympathetic responses to single, 1 s hindlimb muscle contractions in HF‐rEF rats compared to sham rats (Koba et al., 2008). Most importantly, our present findings confirm that the 1 Hz dynamic rat hindlimb muscle stretch and contraction models are valuable experimental tools that may be used to study exaggerated sympathetic/cardiovascular responses to isolated dynamic mechanoreflex activation and EPR activation in human HF‐rEF patients (Amann et al., 2014; Cui et al., 2006, 2011; Middlekauff et al., 2004).

In healthy humans (Cui et al., 2007; Middlekauff & Chiu, 2004) and animals (Stebbins et al., 1986), inhibition of the COX enzyme reduces the sympathetic and cardiovascular responses to exercise/skeletal muscle contraction. The effect of COX inhibition is attributable to, at least in part, an attenuation of the responsiveness of thin fiber muscle afferents to skeletal muscle contraction (Hayes et al., 2006). Several studies have investigated the identity of the receptor(s) on sensory endings of thin fiber muscle afferents for COX metabolites that account for the attenuation of the EPR following COX inhibition. Specifically, inhibition of TxA_2_‐Rs (Leal et al., 2011), but not EP3‐Rs or EP4‐Rs (Stone et al., 2015; Yamauchi et al., 2013), has been shown to reduce the EPR responses to static hindlimb muscle contraction in healthy rats. In contrast to the findings of Leal et al. (2011), in the present investigation we found that inhibition TxA_2_‐R had no effect on the sympathetic or cardiovascular responses to 1 Hz dynamic skeletal muscle contraction. This discrepancy may be attributed to the different modalities employed to elicit hindlimb skeletal muscle contraction (i.e., static vs. dynamic). The possibility of redundancy among the receptors evoking the EPR in health during dynamic contractions must also be considered (Stone et al., 2015).

Previously, we demonstrated in HF‐rEF rats that hindlimb arterial injection of the TxA_2_‐R antagonist daltroban reduced the sympathetic and cardiovascular responses to 1 Hz dynamic skeletal muscle stretch (Butenas, Rollins, et al., 2021), but not static hindlimb skeletal muscle stretch (Butenas et al., 2020). We speculated that those contrasting findings in HF‐rEF rats pertaining to the role played by TxA_2_‐Rs during different mechanoreflex modalities (i.e., static stretch vs. dynamic stretch) were best explained by the fact that these different modalities may activate substantially different classes of mechanically activated channels (for discussion, see: Butenas, Rollins, et al., 2021). We therefore concluded that TxA_2_‐Rs on the sensory endings of thin fiber muscle afferents contribute to a chronic sensitization of the mechanically activated channels that underlie dynamic/rhythmic mechanoreflex activation. We now believe our present findings that TxA_2_‐R blockade with daltroban reduced the sympathetic and cardiovascular responses to 1 Hz dynamic skeletal muscle contraction in HF‐rEF rats likely reflected that chronic sensitization of mechanically activated channels produced by TxA_2_‐Rs. That conclusion is supported by the marked reduction of the RSNA bursts that were synchronized to muscle tension development in HF‐rEF rats following TxA_2_‐R blockade, especially within the first 5 s (see Figure 6). An acute sensitization of mechanically activated channels produced by contraction‐induced elevations in COX metabolites (Hayes et al., 2006; McCord et al., 2008), as well as a small role for TxA_2_‐R in the metaboreflex component of the EPR in HF‐rEF rats is also possible (Leal et al., 2011). If present, those actions may have occurred additively with the chronic sensitization to produce the overall TxA_2_‐R mediated effects in our experiments.

The precise mechanism behind the chronic mechanoreflex sensitization produced by TxA_2_‐Rs in HF‐rEF remains unclear. In the present investigation we found no difference between SHAM and HF‐rEF rats in TxA_2_‐R protein or mRNA expression in lumbar DRG which is an important reproduction of our recent findings (Butenas et al., 2020). Thus, it does not appear that an increased expression of TxA_2_‐Rs on the sensory endings of thin fiber muscle afferents accounts for the TxA_2_‐R‐mediated chronic mechanoreflex sensitization in HF‐rEF. An increased basal concentration of COX products of arachidonic metabolism in HF‐rEF may have offered an alternative explanation for the TxA_2_‐R‐mediated chronic mechanoreflex sensitization. We hypothesized as much based on previous findings showing elevated COX‐2 protein expression within skeletal muscle homogenates of HF‐rEF patients/animals compared with controls (Butenas et al., 2020; Morales et al., 2012; Smith, Hart, et al., 2020). Specifically, we hypothesized that HF‐rEF would result in higher levels of basal COX‐2 metabolites within the white portion of the gastrocnemius muscle compared to SHAM counterparts. We selected this region of the triceps surae muscle as this muscle region has been shown specifically to have elevated COX‐2 protein expression in HF‐rEF rats compared with healthy controls (Butenas et al., 2020; Morales et al., 2012). In contrast to our hypothesis, we found that basal microdialysate fluid concentrations for PGE_2_ and TxB_2_ from the white portion of the gastrocnemius muscle were not different between SHAM and HF‐rEF rats. This is generally consistent with the findings of Scott et al. (2002) who reported no difference in venous effluent concentrations from the resting forearm for PGE_2_ and PGF_1α_ between HF‐rEF patients and age‐matched controls. Those findings of similar COX metabolite concentrations are further supported by our present finding that COX‐2 enzymatic activity within skeletal muscle homogenates from the white portion of the gastrocnemius muscle was not different between SHAM and HF‐rEF rats. Collectively, our findings suggest that the TxA_2_‐R‐mediated chronic mechanoreflex sensitization in HF‐rEF is not attributed to an increased presence of COX metabolites within the skeletal muscle interstitial space. It remains possible, however, that skeletal muscle contraction results in a greater accumulation of COX metabolites within the skeletal muscle interstitial space in HF‐rEF compared to SHAM rats which acts in addition to the chronic mechanoreflex sensitization.

Given we found no difference in TxA_2_‐R expression within lumbar DRG or basal COX metabolites within the skeletal muscle interstitial space between SHAM and HF‐rEF rats, how may we explain the effect of TxA_2_‐R blockade on the sympathetic and cardiovascular responses to hindlimb muscle contraction (present study) and hindlimb muscle stretch (Butenas, Rollins, et al., 2021)? It is possible that the site of amplification of the TxA_2_‐R signaling in HF‐rEF resides within the intracellular pathways within the sensory neuron endings. Although speculative, there is at least some indirect evidence for such a phenomenon. Specifically, it may be that in HF‐rEF there is an amplification of inositol trisphosphate and/or diacylglycerol signaling (the G protein‐coupled signaling pathways associated with TxA_2_‐Rs) within sensory neuron endings. Such an amplification may result in an increased cytosolic calcium concentration within the sensory neuron ending which, in turn, may sensitize mechanically activated channels (Zhuang et al., 2015). Investigation into these intracellular signaling pathways and their possible contribution to the mechanoreflex and EPR in HF‐rEF is warranted.

Several experimental considerations should be noted. First, the EPR is one of the several autonomic control signals that likely contributes to the aberrant sympathetic and cardiovascular adjustments to exercise in HF‐rEF. For example, central command (Koba et al., 2006), the carotid chemoreflex (Li et al., 2006; Machado et al., 2020; Stickland et al., 2007), and the arterial baroreflex (Grassi et al., 1995; Mancia et al., 1992) have all been reported to contribute to aberrant sympathetic and cardiovascular responses during exercise in HF‐rEF. Whether TxA_2_‐Rs contribute to exaggerated increases in SNA during exercise in HF‐rEF patients when all these autonomic control mechanisms are working in concert with one another (i.e., during whole body exercise) remains unknown. Second, RSNA was not measured in the vehicle and systemic control experiments. We elected to perform the vehicle and systemic control protocols in experiments in which technical factors precluded successful RSNA recording in order to maximize the statistical power of the RSNA comparisons in the main experimental protocols. Third, only male rats were used in the present investigation. Future studies are needed to determine the role of TxA_2_‐R signaling in the EPR in female rats with and without HF‐rEF. Lastly, we cannot rule out the possibility that an increase in hindlimb muscle blood flow and consequent washout of metabolites produced during muscle contraction may also have contributed to the attenuated EPR following TxA_2_‐R blockade in rats with HF‐rEF. Although this seems unlikely given that TxA_2_‐R blockade had no effect in SHAM rats.

In summary, we found that the sympathetic and cardiovascular responses to 1 Hz dynamic hindlimb muscle contraction were larger in rats with HF‐rEF compared to the responses in SHAM‐ operated healthy control rats. We also found that the hindlimb arterial injection of a TxA_2_‐R antagonist reduced these exaggerated responses to dynamic hindlimb muscle contraction in HF‐rEF rats. Although the precise mechanism underlying the TxA_2_‐R signaling amplification remains unknown, these data suggest that TxA_2_‐R signaling within the sensory endings of thin fiber muscle afferents contribute to the exaggerated EPR evoked during dynamic muscle contractions in HF‐rEF. Future studies should seek to determine the precise mechanism underlying the TxA_2_‐R signaling amplification. Our findings may have important implications for our understanding of the reflex control of the sympathetic nervous system during exercise in the over 26 million people worldwide with HF‐rEF (Ponikowski et al., 2014).

## CONFLICT OF INTEREST

The authors have no conflict of interest.

## AUTHOR CONTRIBUTIONS

A.L.E.B., K.S.R., C.J.A., T.I.M., and S.W.C.: conception and design of the study; A.L.E.B., K.S.R., A.C.W., S.K.P., S.T.H., C.J.A., K.S.H., T.I.M., and S.W.C.: data acquisition, analysis, and interpretation. A.L.E.B and S.W.C.: drafting of the article. All authors contributed to manuscript editing and approved the final version.
